# Umbilical cord blood: a comprehensive review of protective and restorative properties in clinical applications – a narrative review

**DOI:** 10.1097/MS9.0000000000003823

**Published:** 2025-08-29

**Authors:** Emmanuel Ifeanyi Obeagu

**Affiliations:** Department of Biomedical and Laboratory Science, Africa University, Mutare, Zimbabwe

**Keywords:** immunomodulation, neuroprotection, regenerative medicine, stem cell therapy, umbilical cord blood

## Abstract

Umbilical cord blood has emerged as a valuable biological resource rich in hematopoietic stem and progenitor cells, offering promising therapeutic potential in regenerative medicine, hematologic disorders, and immune modulation. Compared to bone marrow and peripheral blood stem cells, UCB demonstrates several clinical advantages, including lower risk of graft-versus-host disease (GVHD), increased tolerance for human leukocyte antigen (HLA) mismatch, and rapid availability. Transplantation success rates with UCB have improved significantly, with recent studies reporting overall survival rates of 60–70% in pediatric hematopoietic stem cell transplant recipients and 55–65% in adult recipients, particularly in malignant conditions. The incidence of acute GVHD following UCB transplantation ranges from 20% to 40%, while chronic GVHD occurs in approximately 10–20% of cases – lower than rates observed with other stem cell sources. Moreover, UCB-derived stem cells are being investigated for their regenerative and immunomodulatory capabilities in conditions such as cerebral palsy, type 1 diabetes, and ischemic injury, with early-phase trials showing encouraging safety and efficacy profiles. Despite these advancements, disparities in cost-effectiveness and accessibility remain pressing issues. Public cord blood banks offer greater equity in access and have facilitated most unrelated transplants, whereas private banks, often costly, primarily serve families for autologous use with limited clinical indication. This review provides a comprehensive analysis of the biological underpinnings, clinical applications, and outcomes associated with UCB-based therapies, while highlighting ongoing challenges in global access, standardization, and therapeutic scalability.

## Introduction

Umbilical cord blood (UCB), once considered medical waste, has emerged as a clinically valuable source of hematopoietic stem cells (HSCs), mesenchymal stromal cells (MSCs), immune effector cells, and bioactive molecules. Since the first successful UCB transplantation in 1988 for a child with Fanconi anemia, it has transformed therapeutic strategies in hematology, oncology, regenerative medicine, and immunotherapy. Globally, more than 40 000 UCB transplants have been performed, supported by over 800 000 publicly banked units, underscoring its integration into mainstream clinical practice^[1–3]^. Biologically, UCB is characterized by a high frequency of primitive CD34⁺ HSCs – ranging from 1 to 5 × 10^6^ cells/mL – exhibiting robust proliferative capacity, longer telomeres, and reduced immunological maturity compared with bone marrow (BM) and peripheral blood sources. MSCs derived from UCB contribute to tissue repair via paracrine signaling, modulation of inflammatory cascades, and secretion of angiogenic factors such as vascular endothelial growth factor (VEGF) and transforming growth factor-β (TGF-β). UCB also contains exosomes enriched with microRNAs that influence critical regenerative pathways, including PI3K/Akt and NF-κB signaling^[4–6]^.

**HIGHLIGHTS**

- Umbilical cord blood (UCB) offers high proliferative potential with lower GVHD risk.
- Median neutrophil engraftment is ~24 days; platelet ~35 days.
- Exosomes modulate PI3K/Akt and NF-κB pathways.
- Public banking is cost-effective but underutilized; private banking raises ethical concerns.
- UCB integration with CAR-T and gene editing holds future clinical promise.

**Figure 1.**
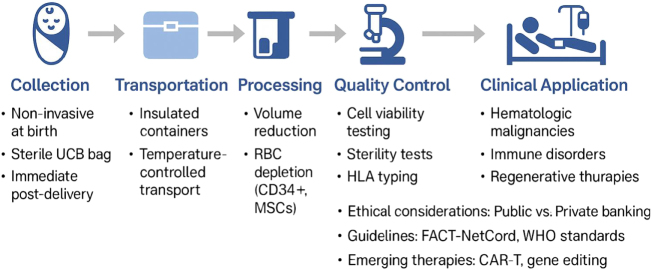
From collection to clinical application.

Clinically, UCB offers distinct advantages: rapid and safe collection, lower risk of graft-versus-host disease (GVHD), and feasibility of partial HLA matching, which expands access for ethnically diverse patient populations. Median rates of acute GVHD after UCB transplantation are approximately 20–30%, compared with 40–50% for BM or peripheral blood stem cell (PBSC) sources, without compromising graft-versus-leukemia effects. Beyond hematopoietic reconstitution, emerging studies support its role in neuroprotection, immune tolerance induction, and cardiac and metabolic tissue repair^[7–9]^. Despite these benefits, challenges persist – including limited cell dose per unit, slower engraftment kinetics, and cost/accessibility disparities between public and private banking models. Positions from the American Academy of Pediatrics (AAP) and the World Health Organization (WHO) recommend prioritizing public banking to maximize equitable access while recognizing potential autologous applications in select cases (Fig. 1)[10]. The aim of this narrative review is to synthesize current evidence on the biological composition of UCB, elucidate its protective and restorative mechanisms, evaluate its clinical applications across diverse specialties, and explore future innovations that may expand its therapeutic scope.

## Aim and review methods

This narrative review aims to comprehensively examine the therapeutic potential, biological attributes, and clinical applications of UCB, with emphasis on its immunomodulatory properties, transplantation outcomes, and emerging innovations. Particular attention is given to comparative analyses with other stem cell sources, including BM and peripheral blood, as well as the ethical, economic, and regulatory dimensions of UCB banking.

The review was conducted using a thematic approach rather than a systematic protocol, as it does not adhere to Preferred Reporting Items for Systematic Reviews and Meta-Analyses (PRISMA) guidelines. Literature was identified through structured searches in databases including PubMed, Scopus, and Web of Science using keywords such as “umbilical cord blood,” “stem cell transplantation,” “GVHD,” “engraftment,” “exosomes,” and “banking.” Priority was given to peer-reviewed articles, clinical trial reports, and authoritative guidelines from international bodies such as the WHO, the AAP, and FACT-NetCord. Conflicting evidence was thematically compared, with acknowledgment of heterogeneity in study design, sample size, and methodology where applicable. Key quantitative and mechanistic data were extracted to support synthesis and inform clinical and translational relevance.

## Methods

This article was conducted as a narrative review, designed to synthesize current knowledge on the biological composition, protective mechanisms, and clinical applications of UCB, as well as to explore future therapeutic innovations. Unlike systematic reviews, which employ rigid inclusion frameworks and meta-analytical synthesis, this narrative review uses a flexible thematic approach to integrate diverse study types, including clinical trials, observational studies, preclinical experiments, policy papers, and expert consensus guidelines.

### Literature search strategy

A comprehensive literature search was performed in PubMed, Scopus, Web of Science, and Google Scholar from January 1990 to June 2025. The following keywords and Boolean combinations were used: “umbilical cord blood,” “hematopoietic stem cell transplantation,” “mesenchymal stromal cells,” “immunomodulation,” “neuroprotection,” “regenerative medicine,” and “clinical applications.” Reference lists of relevant articles were also screened to identify additional sources.

### Inclusion and exclusion criteria

Studies were included if they:

- reported original data, systematic reviews, meta-analyses, or authoritative position statements related to UCB biology, transplantation, or therapeutic applications.
- were published in peer-reviewed journals in English.
- included quantitative or qualitative outcome data relevant to UCB’s protective or restorative roles.

The exclusion criteria included:

- conference abstracts without peer-reviewed full texts.
- non-English publications without reliable translations.
- studies with insufficient methodological detail or lacking relevance to the review scope.

### Data extraction and thematic synthesis

Relevant data were extracted on:

- Biological composition (e.g., CD34⁺ cell counts, MSC yields, cytokine profiles, and exosome contents).
- Mechanistic insights (e.g., signaling pathways, immunomodulatory factors, and regenerative mediators).
- Clinical outcomes (e.g., engraftment rates, GVHD incidence, survival data, and functional recovery measures).
- Challenges and limitations (e.g., cell dose constraints, banking access, and engraftment delays).

Thematic synthesis was employed to organize findings into five key domains: (1) biological characteristics of UCB, (2) protective mechanisms, (3) restorative clinical applications, (4) advantages over other stem cell sources, and (5) challenges with future directions. Conflicting evidence was presented in context, noting differences in study design, patient population, and follow-up duration.

## Quality considerations

While formal risk-of-bias assessment tools were not applied – consistent with narrative review methodology – greater weight was given to large-scale clinical trials, meta-analyses, and consensus guidelines. Preclinical and early-phase studies were included for emerging therapies but discussed with appropriate caution regarding translational limitations.

## Handling of conflicting evidence

In conducting this narrative review, we encountered studies with conflicting findings regarding the efficacy, cellular properties, and clinical applications of UCB-derived stem cells. To address this, contradictory evidence was thematically compared and discussed within the context of study design, sample size, methodological differences, and clinical endpoints. Rather than excluding discordant results, we deliberately highlighted them to provide a balanced overview of the field. Heterogeneity in outcomes – particularly regarding transplantation success rates, GVHD incidence, and the long-term functionality of UCB-derived cells – was acknowledged and critically analyzed. Where appropriate, we noted the influence of geographic, technological, and regulatory variations on reported outcomes. This approach aligns with best practices in narrative synthesis, fostering transparency while reflecting the evolving and multifaceted nature of stem cell research.

## Composition of UCB

UCB is a rich and heterogeneous source of hematopoietic and non-hematopoietic stem cells, immune cells, and progenitor populations. Among its most clinically relevant constituents are hematopoietic stem and progenitor cells (HSPCs), typically identified by CD34⁺ surface expression. The concentration of CD34⁺ cells in UCB varies depending on gestational age, collection volume, and processing method, with typical ranges between 1 and 5 × 10^6^ CD34⁺ cells per unit. These values are generally sufficient for transplantation in pediatric recipients, though may require double-unit transplants or *ex vivo* expansion for adult patients^[11–13]^. UCB also contains MSCs, albeit at a lower frequency than BM. MSC isolation from UCB is less efficient, with success rates ranging from 30% to 60% depending on collection timing and processing protocol. While the absolute yield of MSCs per unit volume is lower than that from BM, UCB-derived MSCs demonstrate favorable biological characteristics, including longer telomere lengths, higher proliferative potential, and reduced senescence, which can enhance their therapeutic utility^[14–16]^. When compared to BM, UCB generally offers a higher ratio of primitive progenitor cells relative to volume, though total nucleated cell and MSC counts are typically lower. Nonetheless, UCB-derived cells exhibit enhanced expansion capacity *in vitro* and greater plasticity, making them particularly attractive for regenerative medicine and immune-modulatory applications (Tables 1 and 2)[17].

**Table 1:** Major components of umbilical cord blood, their functions, and therapeutic benefits

Component	Function	Therapeutic benefits
Hematopoietic stem cells	Self-renewal and differentiation into blood lineages	Used in hematologic malignancies, immune deficiencies, and bone marrow failure syndromes
Mesenchymal stem cells	Support hematopoiesis, immunomodulation, tissue repair	Anti-inflammatory therapies, regenerative medicine (e.g., cardiac, neural repair)
Endothelial progenitor cells	Promote vascular regeneration and angiogenesis	Cardiovascular repair and ischemic tissue recovery
Regulatory T cells (Tregs)	Suppress immune response and prevent autoimmunity	Graft-versus-host disease prevention, autoimmune disease modulation
Natural killer (NK) cells	Innate immune cytotoxicity against tumor and virally infected cells	Cancer immunotherapy, antiviral responses
Exosomes (extracellular vesicles)	Intercellular signaling, miRNA/protein cargo delivery	Modulate inflammation, promote tissue repair, target PI3K/Akt and NF-κB pathways
Cytokines (e.g., IL-10, TGF-β)	Mediate immune suppression and tissue remodeling	Anti-inflammatory effects, enhancement of graft tolerance
Soluble HLA-G and Fas ligand	Induce immune tolerance and inhibit cytotoxic lymphocyte activity	Immune evasion and tolerance in transplantation

IL-10, interleukin-10; TGF-β, transforming growth factor-β.

**Table 2:** Comparative characteristics of UCB, bone marrow, and PB for stem cell transplantation

Parameter	UCB	Bone marrow	PB
CD34⁺ cell count	1–5 × 10^6^ cells/unit	2–10 × 10^6^ cells/kg recipient weight	2–5 × 10^6^ cells/kg recipient weight
Mesenchymal stem cell yield	Low; requires expansion	Moderate to high	Very low
Hematopoietic stem cell frequency	High per unit volume	Lower compared to UCB per volume	High in mobilized PB
Telomere length	Longer	Intermediate	Shorter
Oct4 and stemness marker expression	High	Moderate	Low
Immunogenicity	Low (naïve T cells)	Moderate	High
Graft-versus-host disease	Lower incidence	Moderate to high	Highest
Time to engraftment (neutrophils)	20–30 days	14–21 days	10–14 days
Time to engraftment (platelets)	30–60 days	21–35 days	14–21 days
Collection invasiveness	Noninvasive (postpartum)	Invasive (requires anesthesia)	Minimally invasive (apheresis)
Donor matching requirements	Less stringent (more mismatch tolerated)	HLA-matching required	HLA-matching required
Storage	Cryopreserved	Fresh use or short-term storage	Requires mobilization and immediate use
Common use cases	Pediatrics, rare diseases, research	Allogeneic transplants	Autologous or allogeneic transplants

HLA, human leukocyte antigen; PB, peripheral blood; UCB, umbilical cord blood.

## Protective and immunomodulatory effects of UCB

UCB is a biologically complex and therapeutically versatile tissue, composed of diverse cellular and molecular constituents that collectively underpin its clinical potential. At its core, UCB contains hematopoietic stem and progenitor cells (HSCs), identifiable by CD34⁺ surface expression, with typical yields ranging from 1 to 5 × 10^6^ CD34⁺ cells per milliliter. These HSCs display higher proliferative capacity, longer telomeres, and a greater proportion of primitive phenotypes compared with BM or peripheral blood (PB) stem cell sources. Such features contribute to their capacity for rapid expansion and multilineage differentiation^[18,19]^. Beyond hematopoiesis, UCB harbors mesenchymal stromal cells (MSCs), although at lower frequencies than in BM, with reported yields of 10^2^–10^3^ colony-forming units per collection. These cells exert potent immunomodulatory effects through paracrine signaling and secrete anti-inflammatory cytokines, angiogenic mediators such as VEGF, and extracellular vesicles enriched with regenerative microRNAs. In addition, endothelial progenitor cells (EPCs) – important for angiogenesis and vascular repair – are present, further expanding UCB’s regenerative repertoire^[20,21]^.

The immune cell population of UCB reflects its developmental immaturity, characterized by a higher proportion of naïve T lymphocytes (CD45RA⁺) and reduced memory T cells, alongside natural killer (NK) cells with robust cytotoxic potential. Regulatory T cells (Tregs), expressing CD4⁺CD25⁺FOXP3⁺, contribute to its tolerogenic profile, reducing the incidence and severity of GVHD compared to other stem cell sources^[22,23]^. Molecularly, UCB contains a rich milieu of soluble factors, including interleukin-10, TGF-β, and anti-apoptotic proteins that attenuate inflammatory injury. Exosomes and microvesicles derived from UCB cells carry bioactive cargos – particularly microRNAs targeting PI3K/Akt and NF-κB signaling pathways – which play roles in promoting cell survival, reducing oxidative stress, and stimulating tissue regeneration[24]. When compared with BM and PB stem cells, UCB offers unique biological advantages: higher proliferative index, greater HLA mismatch tolerance, and an inherently lower risk of transmitting latent viral infections. A comparative perspective shows that while UCB collections typically yield smaller absolute cell doses than adult stem cell harvests, the immunological immaturity and functional potency of these cells often compensate for this limitation in both hematopoietic and non-hematopoietic applications (Table 1)^[25,26]^.

## Restorative potential and clinical applications of UCB

The restorative potential of UCB extends beyond its established role in hematopoietic reconstitution, encompassing applications in regenerative medicine, immune modulation, and organ repair. Clinically, UCB transplantation has become a standard therapeutic option for hematological malignancies, BM failure syndromes, and a growing number of inherited metabolic and immunodeficiency disorders. Its unique immunobiological profile – characterized by naïve lymphocyte predominance, regulatory T cell enrichment, and reduced alloreactivity – facilitates engraftment even with partial HLA matching, broadening donor availability^[27,28]^. In hematology and oncology, UCB transplantation achieves complete hematopoietic reconstitution in patients lacking matched BM donors. Median neutrophil engraftment occurs within 21–28 days, with platelet recovery in 35–45 days. Long-term survival rates in pediatric acute lymphoblastic leukemia exceed 60–70%, with lower chronic GVHD incidence compared to PBSC transplants. However, delayed engraftment and cell dose limitations remain key challenges, particularly in adults, prompting the use of double-unit transplantation and *ex vivo* expansion strategies^[29,30]^.

In neurological disorders, early phase clinical trials have demonstrated functional improvements in conditions such as cerebral palsy, hypoxic-ischemic encephalopathy, and traumatic brain injury. The proposed mechanisms involve neurotrophic factor secretion, reduction of neuroinflammation, and promotion of neural plasticity. For example, in a phase II trial for pediatric cerebral palsy, autologous UCB infusion was associated with measurable gains in gross motor function over 12 months. Yet, not all studies have shown significant benefit, highlighting the need for larger, controlled trials to define patient selection and optimal dosing[31]. Cardiovascular repair is another emerging application, leveraging UCB-derived EPCs and paracrine mediators to promote angiogenesis and myocardial recovery. In pilot studies of ischemic cardiomyopathy, intracoronary infusion of UCB mononuclear cells resulted in modest improvements in left ventricular ejection fraction (mean increase 3–5%) and functional capacity. Preclinical models suggest that these effects may be mediated by EPC incorporation into neovessels and secretion of pro-angiogenic factors[32].

In autoimmune and metabolic diseases, UCB has shown promise in modulating immune dysregulation and preserving residual tissue function. Trials in type 1 diabetes have explored autologous UCB infusions to preserve pancreatic β-cell activity, with mixed outcomes depending on disease stage at intervention. Similarly, exploratory studies in multiple sclerosis and systemic lupus erythematosus report reduced inflammatory markers and improved clinical scores following UCB-derived cell therapy, although larger confirmatory studies are required[33]. Beyond these targeted indications, UCB is under investigation in liver disease, lung injury, and tissue engineering. In chronic liver injury models, UCB-derived MSCs attenuate fibrosis and stimulate hepatocyte regeneration, while in pulmonary injury, they reduce inflammation and promote epithelial repair. Advances in bioengineering are combining UCB cells with biomaterial scaffolds for organ and tissue reconstruction, potentially expanding its therapeutic reach[34].

## Advantages of UCB over other stem cell sources

UCB offers a constellation of advantages over traditional stem cell sources such as BM and PBSCs, rooted in its unique biological, logistical, and immunological characteristics. One of the most compelling benefits is rapid and safe collection. UCB is obtained immediately after birth via a noninvasive procedure that poses no risk to the mother or newborn, in contrast to BM harvesting, which requires anesthesia and carries potential complications such as pain, infection, and bleeding^[35,36]^. From a biological perspective, UCB contains a higher proportion of primitive hematopoietic stem cells with longer telomeres and greater proliferative potential compared with adult stem cell sources. This intrinsic proliferative advantage facilitates durable engraftment despite lower absolute cell doses per unit. Immunologically, UCB cells are predominantly naïve T lymphocytes (CD45RA⁺) and exhibit lower expression of co-stimulatory molecules, enabling greater tolerance for HLA mismatches. This property significantly increases the likelihood of finding a suitable donor, especially for patients from ethnically diverse or underrepresented populations^[37,38]^.

Clinical outcomes further highlight these immunological benefits. The incidence of acute GVHD after UCB transplantation is approximately 20–30%, compared to 40–50% for PBSC transplants, while chronic GVHD rates are similarly reduced. Importantly, the graft-versus-leukemia effect remains preserved, maintaining relapse protection in malignant disease^[39,40]^. Logistically, availability and banking infrastructure offer distinct advantages. Public cord blood banks maintain ready-to-ship, cryopreserved units that can be accessed within days – a critical factor for patients requiring urgent transplantation. In contrast, BM and PBSC collection depends on donor scheduling, availability, and health status, which may delay treatment^[41,42]^. UCB is also associated with a lower risk of transmissible infections, particularly latent viruses such as cytomegalovirus, due to the short lifespan and limited antigen exposure of the newborn’s immune system. Processing methods in accredited banks (FACT and American Association of Blood Banks (AABB)) include rigorous sterility testing, further minimizing contamination risk[5]. While engraftment kinetics are slower – median neutrophil recovery occurring in 21–28 days for UCB compared to 14–21 days for PBSCs – ongoing innovations such as double-unit transplantation, *ex vivo* expansion with small molecules like UM171, and co-infusion with accessory cells are closing this gap. Moreover, the reduced GVHD risk often offsets the transient period of increased infection susceptibility associated with delayed engraftment^[43,44]^.

## Mechanistic basis for the superior plasticity and proliferative potential of UCB-derived stem cells

UCB-derived stem cells exhibit superior plasticity and proliferative capacity compared to their adult counterparts, making them an attractive source for regenerative and hematopoietic therapies. This enhanced potential is underpinned by several intrinsic biological characteristics. One of the key mechanisms is longer telomere length in UCB-derived HSPCs. Telomeres, repetitive nucleotide sequences at the ends of chromosomes, serve as protective caps that preserve chromosomal integrity during cell division. In UCB cells, telomeres are significantly longer than those in adult BM or peripheral blood-derived stem cells, which allows for extended replicative potential and reduced senescence during *ex vivo* expansion or post-transplantation proliferation[45].

Another defining feature is the elevated expression of pluripotency-associated transcription factors, particularly Octamer-binding transcription factor 4 (Oct4). Oct4 plays a critical role in maintaining the undifferentiated state and self-renewal capacity of stem cells. Higher Oct4 expression in UCB stem cells suggests a retained developmental plasticity reminiscent of embryonic stem cells, enhancing their capacity to differentiate into a broader range of cell types under appropriate cues[46]. Furthermore, epigenetic signatures in UCB stem cells contribute to their enhanced functional attributes. Notably, UCB-derived HSPCs exhibit lower levels of global DNA methylation, particularly at promoter regions of genes involved in self-renewal, lineage commitment, and cell cycle regulation. Hypomethylation in these regions facilitates open chromatin conformations, allowing for more dynamic gene expression changes in response to environmental signals. This epigenetic flexibility is thought to prime UCB cells for rapid proliferation and differentiation when transplanted or exposed to regenerative stimuli[26].

## Public versus private UCB banking: ethical, economic, and accessibility perspectives

The debate over public versus private UCB banking remains a prominent issue in perinatal care, stem cell policy, and bioethics. While both banking models serve valuable roles in clinical practice, their implications differ significantly in terms of accessibility, equity, and societal benefit[44]. Public UCB banks collect and store donated cord blood for unrelated use, making units available to any compatible patient in need of HSCT. These banks operate under national or regional health care systems and prioritize equitable distribution. Private banks, on the other hand, charge a fee to collect and store cord blood exclusively for potential future use by the donor child or family, often marketed as a form of biological insurance[45]. The AAP firmly supports public banking, stating in its policy guidelines that “the chances of a child needing their own banked cord blood are extremely small,” estimated at 1 in 1000 to 1 in 200 000. The AAP advises against routine private banking unless there is a known family history of a disease treatable with HSCT. It emphasizes that public donation is a more socially responsible and medically effective use of cord blood, contributing to a global pool that benefits a wider patient population[46].

Similarly, the WHO and World Marrow Donor Association advocate for the expansion of public cord blood banks, citing the urgent need to diversify donor registries to increase the likelihood of finding HLA-matched units, especially for ethnic and racial minorities who are underrepresented in current inventories. WHO also warns that aggressive marketing by private banks may mislead families into banking for speculative or unproven therapies[26]. From an economic standpoint, private cord blood banking is associated with substantial upfront and annual maintenance costs, which can range from $1000 to $2500 initially, plus $100–$200 per year in storage fees. These costs pose a barrier to access, particularly in low- and middle-income countries and raise concerns about health care inequity. In contrast, public banking is usually free of charge to donors and funded by government health agencies or philanthropic sources[47]. Ethically, public banking aligns more closely with principles of justice and beneficence by contributing to a shared resource for the common good. Critics of private banking argue that it commodifies biological materials and may exploit parental fears. Furthermore, the limited clinical utility of autologous cord blood in many conditions – due to the presence of genetic mutations in the harvested cells – calls into question the cost-effectiveness of private storage for most families[48].

## Challenges in the clinical application of UCB

Despite its remarkable therapeutic promise, the clinical application of UCB is not without limitations. One of the most prominent challenges is the limited cell dose obtainable from a single cord, which may be insufficient for treating adults or larger pediatric patients. This issue can lead to delayed hematopoietic recovery and a higher risk of engraftment failure. Although double UCB transplantation has been introduced to address this limitation, it increases procedural complexity and cost[45]. Another key obstacle is delayed immune reconstitution following UCB transplantation. Compared to BM or PBSCs, UCB stem cells are immunologically immature, resulting in slower immune recovery and an increased susceptibility to infections in the early post-transplant period. While this reduced immune maturity lowers the risk of GVHD, it simultaneously prolongs the patient’s vulnerability to opportunistic pathogens[46]. The availability and quality of stored UCB units also remain pressing concerns. High-quality cryopreservation requires stringent protocols, skilled personnel, and costly infrastructure, which may be limited in resource-constrained settings. Additionally, not all collected units meet the required cell count or viability thresholds, leading to wastage and reduced availability of clinically usable samples[26].

From a biological standpoint, HLA matching is another hurdle. Although UCB transplantation tolerates greater HLA mismatch than BM, mismatching beyond certain thresholds can still compromise engraftment success and increase transplant-related mortality. Furthermore, long-term safety data for some regenerative applications are still evolving, making clinicians cautious about broader adoption[47]. Ethical and regulatory issues also influence UCB’s clinical translation. Debates persist regarding private versus public banking, equitable access to stored units, and the potential commercialization of a resource that is often considered a public good. These considerations underscore the need for robust policies to balance innovation, accessibility, and ethical stewardship[48].

## Recommendations and future directions

To fully realize the therapeutic potential of UCB, several strategic initiatives and research directions should be prioritized. First, standardization of collection, processing, and storage protocols is essential. Adherence to international guidelines, including FACT-NetCord and AABB standards, ensures high-quality units with consistent cell viability, sterility, and functional potency. Strengthening these standards globally can reduce variability across public and private banks and improve clinical outcomes. Second, expanding public banking infrastructure is crucial to enhancing equitable access. Public cord blood banks enable timely transplantation for patients who lack matched family donors, particularly in ethnically diverse or underrepresented populations. Policymakers should support incentives and educational campaigns to increase donation rates and maximize the clinical utility of stored units[49].

Third, innovative strategies to overcome cell dose limitations warrant continued research. Approaches such as *ex vivo* expansion of hematopoietic stem cells using small molecules like UM171, double-unit transplantation, and co-infusion with accessory cells have shown promise in improving engraftment kinetics and reducing infection risks. Integrating these strategies into routine practice could make UCB transplantation feasible for larger pediatric and adult patients. Fourth, integration with advanced cellular therapies offers new therapeutic horizons. UCB-derived cells are being investigated in combination with Chimeric Antigen Receptor T-cell therapy (CAR-T) therapies, gene editing platforms, and tissue engineering scaffolds, potentially extending their application beyond hematologic indications into neurodegenerative, cardiovascular, and metabolic disorders. Early phase clinical trials exploring these approaches should be rigorously designed and reported to establish safety and efficacy. Finally, robust translational research and long-term follow-up are essential to clarify outcomes in emerging indications such as neuroprotection, autoimmune modulation, and organ regeneration. Comparative studies with BM and PBSC therapies can further define scenarios in which UCB offers distinct advantages. Cost-effectiveness analyses, alongside ethical and regulatory considerations, should inform policy and clinical decision-making, ensuring that UCB therapies are both safe and accessible[49].

## Conclusion

UCB represents a unique and valuable source of stem cells, with immense potential for regenerative medicine, immune therapies, and disease treatment. Over the years, significant strides have been made in understanding the protective, restorative, and immunomodulatory properties of UCB, positioning it as a critical component in cell-based therapies. From its use in hematopoietic stem cell transplantation to its applications in gene therapy, cancer immunotherapy, and tissue regeneration, UCB has already demonstrated clinical success in numerous areas. However, several challenges remain in maximizing its clinical utility. These include the limited cell dose, delayed immune reconstitution, HLA mismatching, and ethical considerations surrounding its collection and use. Despite these obstacles, ongoing research and technological innovations, such as *ex vivo* expansion, gene editing, and personalized medicine, hold the promise of overcoming these limitations and expanding the scope of UCB therapies.

## Data Availability

Not applicable as this is a narrative review.

## Research registration unique identifying number (UIN)

Not applicable as this is a narrative review.
